# Patients with Treatment-Requiring Chronic Graft versus Host Disease after Allogeneic Stem Cell Transplantation Have Altered Metabolic Profiles due to the Disease and Immunosuppressive Therapy: Potential Implication for Biomarkers

**DOI:** 10.3389/fimmu.2017.01979

**Published:** 2018-01-24

**Authors:** Håkon Reikvam, Ida-Sofie Grønningsæter, Knut Anders Mosevoll, Roald Lindås, Kimberley Hatfield, Øystein Bruserud

**Affiliations:** ^1^Section Hematology, Department of Medicine, Haukeland University Hospital, Bergen, Norway; ^2^Department of Clinical Science, University of Bergen, Bergen, Norway

**Keywords:** metabolomics, chronic graft versus host disease, stem cell transplantation, biochemical profiling, biomarkers

## Abstract

Chronic graft versus host disease (cGVHD) is a common long-term complication after allogeneic hematopoietic stem cell transplantation. The objective of our study was to compare the metabolic profiles for allotransplant recipients and thereby identify metabolic characteristics of patients with treatment-requiring cGVHD. The study included 51 consecutive patients (29 men and 22 women; median age: 44 years, range: 15–66 years) transplanted with peripheral blood stem cells derived from human leukocyte antigen-matched family donors. All serum samples investigated by global metabolomic profiling were collected approximately 1 year posttransplant (median 358 days). Thirty-one of the 51 patients (61%) had cGVHD 1 year posttransplant. The affected organs were (number of patients) liver/bile duct (23), eyes (15), gastrointestinal tract (14), skin (13), mouth (10), lungs (3), and urogenital tract (1). We compared the metabolic profile for patients with and without cGVHD, and a Random Forrest Classification Analysis then resulted in 75% accuracy in differentiating the two groups. The 30 top-ranked metabolites from this comparison included increased levels of bile acids, several metabolites from the cytokine-responsive kynurenine pathway for tryptophan degradation, pro-inflammatory lipid metabolites, phenylalanine and tyrosine metabolites derived from the gut microbial flora, and metabolites reflecting increased oxidative stress. However, nine of these 30 top-ranked metabolites were probably altered due to cyclosporine or steroid treatment, and we therefore did a hierarchical clustering analysis including all 51 patients but only based on the other 21 cGVHD-specific metabolites. This analysis identified three patient subsets: one cluster included mainly patients without cGVHD and had generally low metabolite levels; another cluster included mainly patients with cGVHD (most patients with at least three affected organs) and high metabolite levels, and the last intermediate group including cGVHD patients with limited organ involvement. We conclude that allotransplant recipients with cGVHD have an altered metabolic profile caused both by the disease and its immunosuppressive treatment.

## Introduction

Allogeneic hematopoietic stem cell transplantation (allo-HSCT) is used in the treatment of severe bone marrow failure and aggressive hematological malignancies, including acute leukemia (1, 2). The treatment approach depends on the ability of the engrafting immune system to remove residual leukemia cells *via* a graft-versus-leukemia effect (1). Allo-HSCT is then a potentially curative treatment, although at the same time the treatment is associated with a relatively high risk of morbidity and mortality due to severe transplant-related complications (3). Chronic graft versus host disease (cGVHD) is then the most common cause of late non-relapse mortality (4–6). Guidelines for the diagnosis and treatment of this complication have recently been published (7). However, the complex immunopathology of cGVHD is still poorly understood (8), and preclinical models have weakness and limitations in the study of the disease (9). An increasing interest for biomarkers, to confirm diagnosis and prognosis in cGVHD, has evolved the last decade (10–13), although still no biomarkers are established in routine clinical practice (10, 13). Among the risk factors for cGVHD are older patient age, previous acute GVHD (aGVHD), reduced intensity conditioning, female donor to male recipient, peripheral blood stem cell (PBSC) grafts and human leukocyte antigen (HLA) mismatched donors (14–19).

Graft versus host disease can be considered an exaggerated manifestation of normal inflammatory mechanisms in which donor lymphocytes encounter foreign antigens in a pro-inflammatory milieu, and this inflammation involves several donor immunocompetent cell subsets (8, 9, 20–22). Metabolic regulation is important for immunoregulation, and we have previously demonstrated that pretransplant cytokine profiles as well as the pretransplant metabolic status of allotransplant recipients is associated with a risk of later aGVHD (23–25).

Our present study was initiated to compare patients with and without cGVHD 1 year posttransplant and thereby identify possible associations between the serum metabolic profile, the diagnosis and severity (i.e., organ involvement) of cGVHD requiring systemic immunosuppression, and the effects of this immunosuppressive (i.e., cyclosporine, steroids) on the metabolic profiles in cGVHD patients.

## Materials and Methods

### Patients’ Characteristics

**Figure 1:**
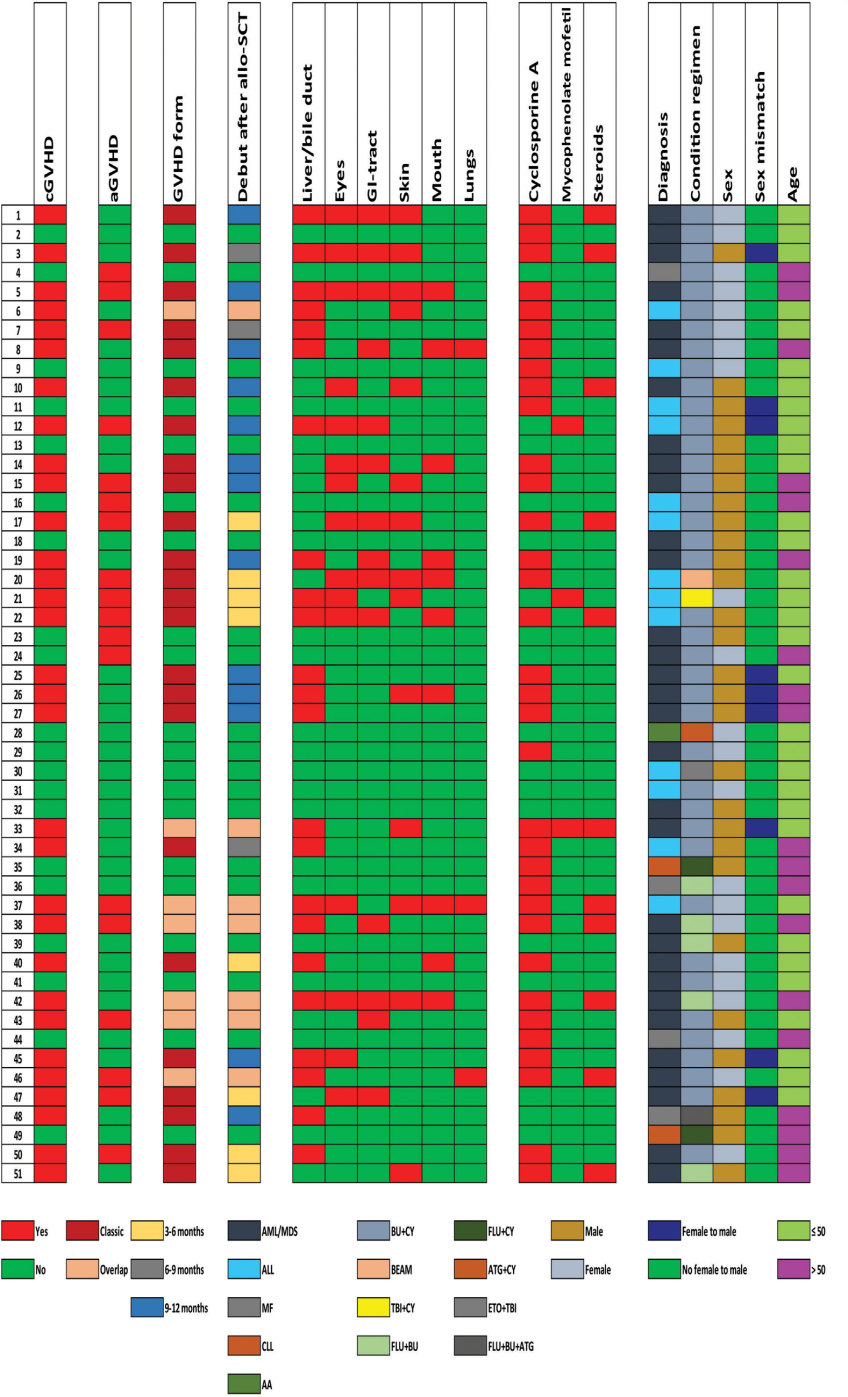
Clinical data of the 51 patients included in the study. The figure presents the clinical and demographical characteristics of the patients, including (from left to right) the presence of chronic graft versus host disease (cGVHD) and acute GVHD, the form of GVHD and the time when developing treatment-requiring cGVHD, organ involvement, ongoing immunosuppressive treatment at the time of sampling, and the clinical characteristics of the patients (hematological diagnosis, conditioning treatment, gender, female to male transplantation, and age). Abbreviations: AML, acute myelogenous leukemia; MDS, myelodysplastic syndrome; ALL, acute lymphoblastic leukemia; CLL, chronic lymphocytic leukemia; MF, myelofibrosis; AA, aplastic anemia; BU, busulfan; CY, cyclophosphamide; ATG, antithymoglobulin; TBI, total body irradiation; ETO, etoposide; FLU, fludarabine.

The study was approved by the local Ethics Committee (Regional Ethics Committee III, University of Bergen, Norway; REK), and the samples were collected after obtaining written informed consent from the patients. The study included 51 consecutive allotransplant recipients (29 men and 22 women; median age: 44 years with range: 15–66 years) with HLA-matched family donors; these patients were transplanted during the period March 2006–December 2014. Ninety-five patients were transplanted in our institution during this period; 25 of them died from treatment-related causes, 6 patients relapsed, and 13 were lost to follow-up. The decision to perform an allo-HSCT was taken by the Norwegian Advisory Board for Stem Cell Transplantation and based on national guidelines. Thus, our study is population-based and includes an unselected and consecutive group of well-characterized patients with family donors. All samples were collected approximately 1 year posttransplant (median 358 days). The patient characteristics are given in Table 1 and Figure 1. Patients were transplanted with granulocyte colony-stimulating factor mobilized PBSC. Most patients received GVHD prophylaxis with cyclosporine A plus methotrexate (*n* = 50), only one patient received cyclosporine A alone.

**Table 1:** Demographical, clinical, and laboratory data for the 51 patients included in the study.

Patient characteristics		Observation	+cGVHD	−cGVHD
**Demographic data and disease history**				
Gender (numbers)	Male/female	29/22	19/12	10/10
Age (years, median and range)		44 (15–66)	44 (18–62)	43 (15–66)
Height (cm, median and range)		172 (149–193)	169 (158–190)	172 (149–193)
Weight (kg, median and range)		69 (42–133)	72 (47–133)	66 (42–98)
BMI (kg/m^2^, median and range)		23.4 (16.9–39.7)	23.7 (17.9–39.7)	22.2 (16.9–28.5)

Diagnosis (numbers)	AML/MDS	31	22	9
	ALL	13	8	5
	CLL	2	0	2
	MF	4	1	3
	AA	1	0	1

Conditioning regimen (numbers)	BU + CY	39	25	14
	ATG + CY	1	0	1
	TBI + CY	1	1	0
	BEAM	1	1	0
	TBI + ETO	1	0	1
	FLU + BU	5	3	2
	FLU + CY	2	0	2
	FLU + BU + ATG	1	1	0

cGVHD organ involvement	Liver (23 patients), eyes (15), gastrointestinal tract (14), skin (13), mouth (10), lungs (3) and urogenital tract (1).			

*Unless otherwise stated the values are presented as median with range given in parenthesis. Height and weight were registered at the start of conditioning therapy*.

*BMI, body mass index; AML, acute myelogenous leukemia; MDS, myelodysplastic syndrome; ALL, acute lymphoblastic leukemia; CLL, chronic lymphocytic leukemia; MF, myelofibrosis; AA, aplastic anemia; BU, busulfan; CY, cyclophosphamide; ATG, anti-thymoglobulin; TBI, total body irradiation; ETO, etoposide; FLU, fludarabine; cGVHD, chronic graft versus host disease*.

Detailed information about individual patients is given in Figure 1, including previous aGVHD, the presence of treatment-requiring cGVHD and when this was diagnosed, the type of cGVHD. All these patients were able to travel with public communication to come to the hospital for controls and blood sampling. All except two patients had active cGVHD requiring continued immunosuppression, but only four patients (patients 37, 38, 42, and 43) had platelet counts observed below 100 × 10^9^/L at the time of sampling (this was also true for the time of diagnosis).

### Diagnosis of cGVHD

Chronic graft versus host disease was diagnosed according to generally accepted criteria based on careful clinical evaluation and additional biopsies for histological confirmation (7, 26).

### Preparation of Serum Samples

All venous blood samples were collected into sterile plastic tubes (BD Vacutainer^®^ SST™ Serum Separation Tubes; Becton-Dickenson, Franklin Lakes, NJ, USA) and allowed to coagulate for 120 min at room temperature (18°C) before centrifugation (300 × *g* for 10 min) and serum collection. All samples were immediately frozen and stored at −70°C until analyzed.

### Analysis of Metabolite Serum Levels

Metabolomic analysis was done in collaboration with Metabolon^®^ (27). Briefly, samples were prepared using the automated MicroLab STAR^®^ system (Hamilton Company, Bonaduz, Switzerland). A recovery standard was added prior to the first step in the extraction process for quality control. To remove protein, dissociate small molecules bound to protein or trapped in the precipitated protein matrix, and recover chemically diverse metabolites, proteins were precipitated with methanol under vigorous shaking for 2 min followed by centrifugation. The resulting extract was divided into four fractions: one for analysis by ultra-performance liquid chromatography-mass spectrometry (UPLC-MS)/MS with positive ion mode electrospray ionization, the second for analysis by UPLC-MS/MS with negative ion mode electrospray ionization, the third for analysis by gas chromatography-mass spectrometry, and the last sample was reserved as a backup. Samples were placed briefly on a Zymark TurboVap^®^ (McKinley Scientific, Sparta, NJ, USA) to remove the organic solvent. The samples for liquid chromatography (LC) were stored overnight under nitrogen before preparation for analysis. For GC, each sample was dried under vacuum overnight before preparation for analysis. A total of 755 metabolites of known identities (named biochemicals) were analyzed in all samples (Table S1 in Supplementary Material).

### Bioinformatical and Statistical Analyses

Bioinformatical analyses were performed using the J-Express (MolMine AS, Bergen, Norway) (28). For hierarchical clustering, all values were median variance standardized and log(2) transformed. The complete linkage was used as the linkage method, and for distance measured the Pearson correlation was used. Statistical analyses were performed using the Statistical Package for the Social Sciences (SPSS) version 15.0 (SPSS Inc., Chicago, IL, USA). The Mann–Whitney *U*-test was used to identify biochemicals that differed significantly between groups. The Chi-Square test was used for analysis of categorized data. Unless otherwise stated *p*-values < 0.05 were regarded as statistically significant.

## Results

### Allotransplant Recipients Are Heterogeneous with regard to Their Serum Metabolic Profile When Tested 1 Year Posttransplant

31 of the 51 patients included in the study (61%) had signs of cGVHD 1 year posttransplant; 29 of the 31 cGVHD patients required systemic immunosuppressive treatment either as prolonged or increased treatment with cyclosporine A (27 patients). Eleven of these 27 cyclosporine-treated patients (22% of the whole cohort) received combination treatment also including systemic steroid therapy, and two additional patients received mycophenolate mofetil monotherapy. The two last patients were diagnosed with cGVHD of the skin and received only topical steroid treatment. Thus, the large majority of the 31 cGVHD patients (29/31) received systemic treatment either as prolonged or increased cyclosporine A therapy, or they received additional immunosuppression with oral prednisolone (daily doses 2.5–40 mg) to maintain disease control. The most commonly affected organ was the liver/bile duct (23 patients; for additional details on organ involvement see Figure 1 and Table 1).

Seven of the 20 patients without signs of cGVHD received prolonged cGVHD prophylaxis at 1 year posttransplant due to either previous severe aGVHD and/or the presence of other risk factors for the development of cGVHD. Thus, a total of 34 patients (67%) received cyclosporine A 1 year posttransplant.

We first used principal component analysis (PCA) and hierarchical clustering to analyze the overall metabolic profiles of the patients. However, these analyses could not distinguish between the 31 patients with and the 20 patients without cGVHD (data not shown); this is probably due to the metabolic heterogeneity for both patient subsets.

### Patients with and without cGVHD Differ in Fatty Acid and Bile Acid Metabolism

**Figure 2:**
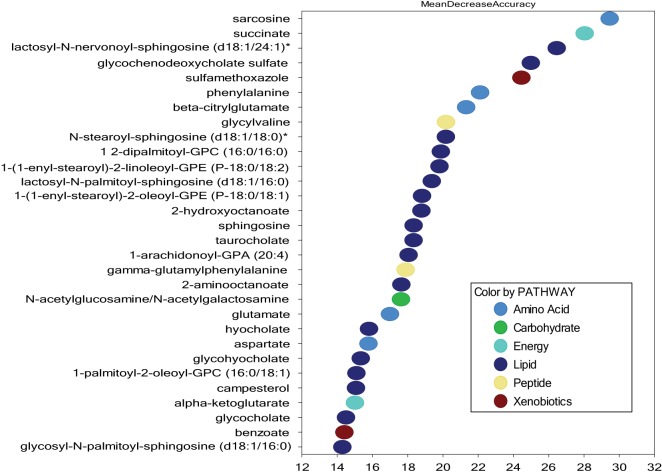
Random forest analysis of the systemic metabolite profiles including all 51 patients; a comparison of patients with and without chronic graft versus host disease (cGVHD) and identification of the 30 top-ranked metabolites showing increased levels in patients with posttransplant cGVHD. Random forest analysis could distinguish between the metabolic signatures of patients with and without acute GVHD with a predictive accuracy of 75.0%. The figure presents the 30 top-ranked metabolites and their classification (indicated in the figure, lower right) based on their importance for the identification of the two patient subsets.

In contrast to PCA and unsupervised hierarchical clustering, random forest analysis is an unbiased and supervised classification technique based on an ensemble of a large number of decision trees. In addition to producing a metric of predictive accuracy (Figure 2), this analysis also gives a list of the metabolites ranked according to their importance for the classification scheme, i.e., their degree of difference between the two compared groups. Random forest analysis of serum metabolic profiles differentiated patients with and without cGVHD with a predictive accuracy of 75%. Eighteen of the 30 top-ranked metabolites from this comparison reflected differences in lipid/fatty acid/bile acid metabolism (Figure 2), most of them belonging to the annotations sphingolipids, plasmalogens/lysoplasmalogens, lysolipids, and phospholipids. We therefore compared the levels of all metabolites from these classes for patients with and without cGVHD. These subclasses include a total of 122 metabolites (Table S1 Supplementary Material), and 46 of them were significantly increased in patients with cGVHD.

**Figure 3:**
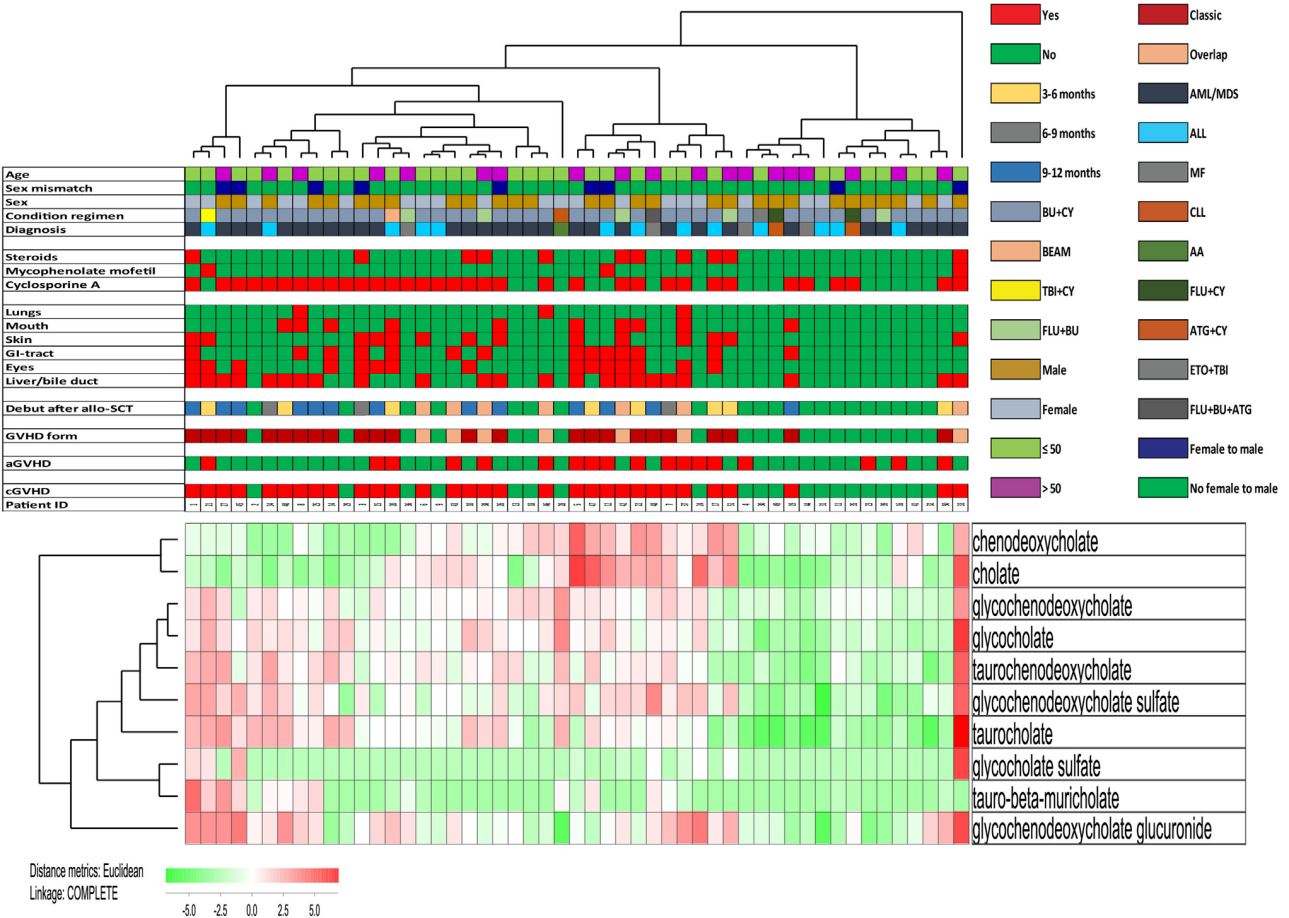
Hierarchical clustering analysis including all 51 patients and based only on 10 primary bile acid metabolites, identification of patient subsets with high frequency of chronic graft versus host disease (cGVHD) patients. We performed a hierarchical clustering analysis (Euclidian Correlation, complete linkage) based on the bile acid metabolites indicated to the right in the figure. The heat map and the corresponding dendrograms are shown in the figure. As indicated to the lower left in the figure red indicates high metabolite levels and green low levels. The clinical characteristics of each individual patient are presented in the upper part of the figure; for the lower horizontal bars the presence of a factor is indicated by red and the absence by green, whereas the color codes for the upper horizontal bars are explained in the figure. We identified two main clusters; the left included a major part of patients with cGVHD, whereas the right cluster included mainly patients without cGVHD. 28 of the 31 cGVHD patients clustered in the group with high bile acid metabolite levels, two patients clustered in the group with low levels, and the last cGVHD patient was an outlier. The frequency of cGVHD patients differed significantly between the two main clusters (Chi-square test, *p* < 0.0001).

Four of the 30 top-ranked metabolites (i.e., the secondary bile acid hyocholate and the primary bile acids glycochenodeoxycholate sulfate, taurocholate, and glycocholate) reflect differences in bile acid metabolism and were increased in patients with cGVHD (Figure 2). The subclass primary bile acids include 10 metabolites, and eight of them were significantly increased in patients with cGVHD. We therefore performed a hierarchical cluster analysis based on the serum levels of the 10 primary bile acid metabolites (Figure 3). This analysis identified a minor subset of 14 patients with generally low levels of these metabolites and including 12 of the 20 patients without cGVHD; only two patients with cGVHD were included and both patients required systemic immunosuppression with only cyclosporine A alone. The remaining patients (a major subset of 36 patients together with one outlier) showed relatively high levels of most primary bile acid metabolites and thus included 28 of the 31 patients with cGVHD, i.e., all patients requiring systemic steroids were included among these 37 patients. The difference in frequency of patients with cGVHD between these two groups was statistically significant (Chi-square test, *p* < 0.0001). Finally, neither the cGVHD patients receiving cyclosporine alone, receiving additional systemic steroids nor having cGVHD with liver involvement clustered together in this analysis.

### A Comparison of Patients with and without cGVHD by Metabolic Pathway Analysis—Increased Levels of Metabolic Markers of Inflammation, Protein Degradation and Oxidative Stress in cGVHD

**Figure 4:**
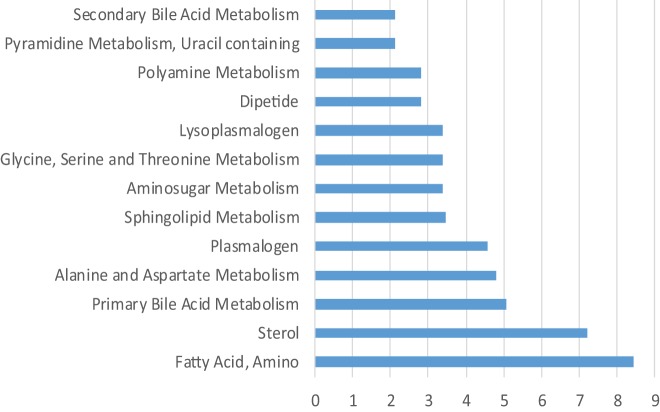
Metabolic pathway analysis based on the analysis of all 51 patients; a comparison of patients with and without chronic graft versus host disease (cGVHD). The figure presents the metabolic pathways with an enrichment score > 2 when comparing patients with and patients without cGVHD.

We next performed a metabolic pathway analysis to compare patients with and without cGVHD (Figure 4). The nine highest ranked metabolic classes included (1) amino fatty acid metabolism (a small class only including two metabolites in our analysis); (2) sphingolipid, plasmalogen, and lysoplasmalogen metabolites; (3) sterol and primary bile acid metabolites; (4) amino acid metabolites (alanine and aspartate, glycine, serine, and threonine); and (5) amino sugar metabolism. Thus, this alternative analysis, which is based on the overall results and not only the highest ranked metabolites, shows that fatty acid/triglyceride/bile acid metabolism differs between patients with and without cGVHD not only when comparing the highest ranked metabolites but also when comparing the overall results.

**Figure 5:**
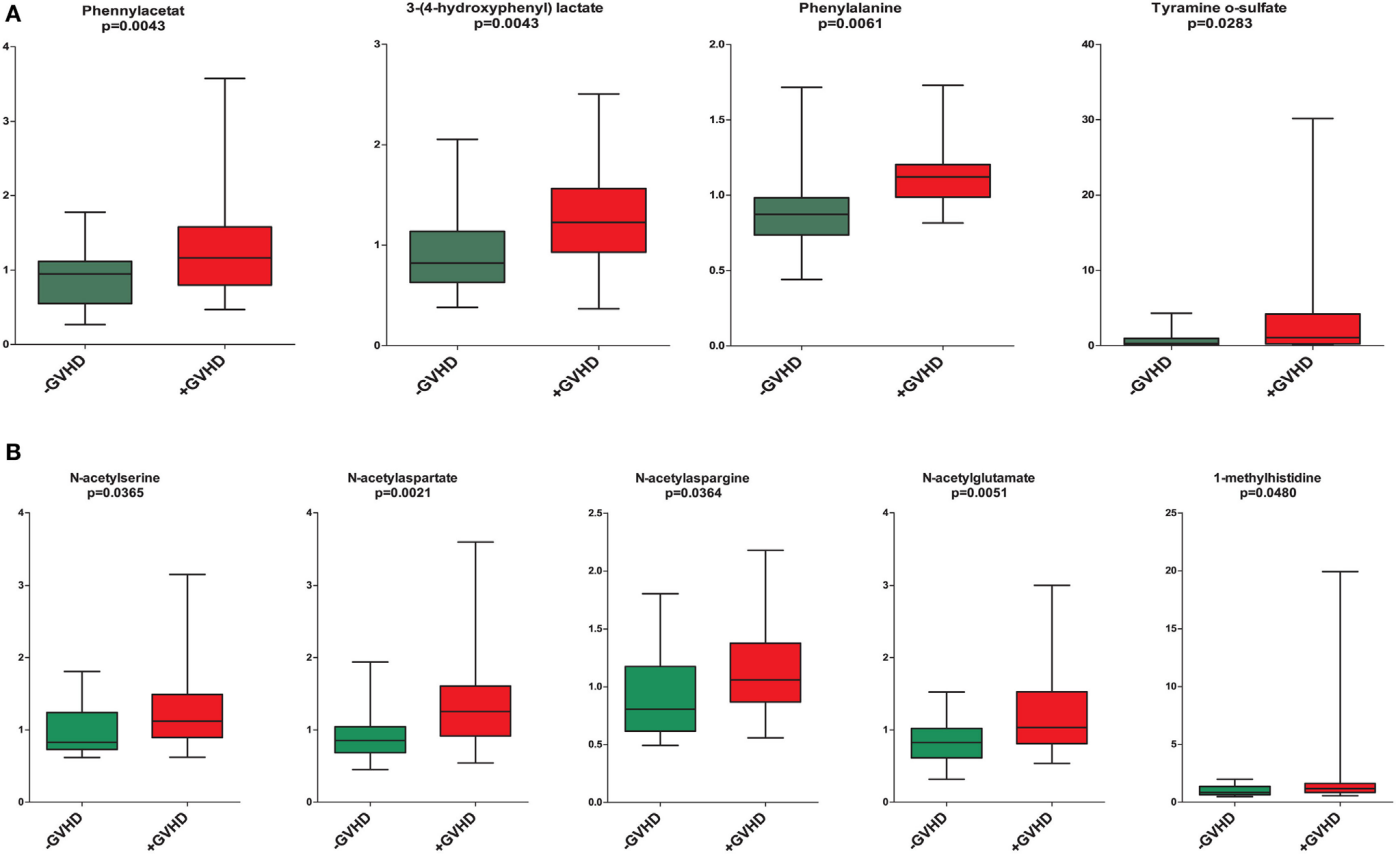
Altered phenylalanine/tyrosine metabolism **(A)** and altered proteolysis **(B)**; a comparison of single metabolites including all patients with and without chronic graft versus host disease (cGVHD). [**(A)**, upper part] The serum levels of the four metabolites phennylacetat, 3-(4-hydroxyphenyl) lactate, phenylalanine, and tyramine *o*-sulfate were significantly increased in cGVHD patients compared to patients without cGVHD. [**(B)**, lower part] The serum levels of the proteolysis markers *N*-acetylserine, *N*-acetylaspartate, *N*-acetylasparagine, *N*-acetylglutamate, and 1-methylhistidine were significantly increased in cGVHD patients (marked with red) compared with patients without cGVHD (marked with green). All results are presented as the median, the 25%/75% percentiles and the variation range; results for cGVHD patients are presented as red boxes whereas patients without cGVHD are marked with green. The metabolites and the corresponding *p*-values (Mann–Whitney *U*-test) are shown at the top of each individual figure.

The presence of cGVHD was associated with a metabolic signature consistent with ongoing inflammation and significantly increased levels of (1) the three lysolipid metabolites 1-linoleoyl-GPC (18:2), 1-oleoyl-GPC (18:1), 1-palmitoleoyl-GPC (16:1), (2) the eicosanoid 12-HETE; and (3) the sphingolipid sphingosine (Figure 2) (29, 30). These signs of inflammation could still be detected despite the systemic immunosuppressive treatment for the large majority of the cGVHD patients. Furthermore, patients with cGVHD showed a significant increase in phennylacetat, 3-(4-hydroxyphenyl) lactate, phenylalanine, and tyramine *o*-sulfate compared with patients without cGVHD; a possible explanation for these differences is altered gastrointestinal function (31), probably caused by gastrointestinal disturbances and altered microbial flora (Figure 5). Furthermore, increased levels of several markers for proteolysis and accelerated protein catabolism were also detected in the cGVHD patients (32), including *N*-acetylserine, *N*-acetylaspartate, *N*-acetylasparagine, *N*-acetylglutamate, and 1-methylhistidine (Figure 5).

**Figure 6:**
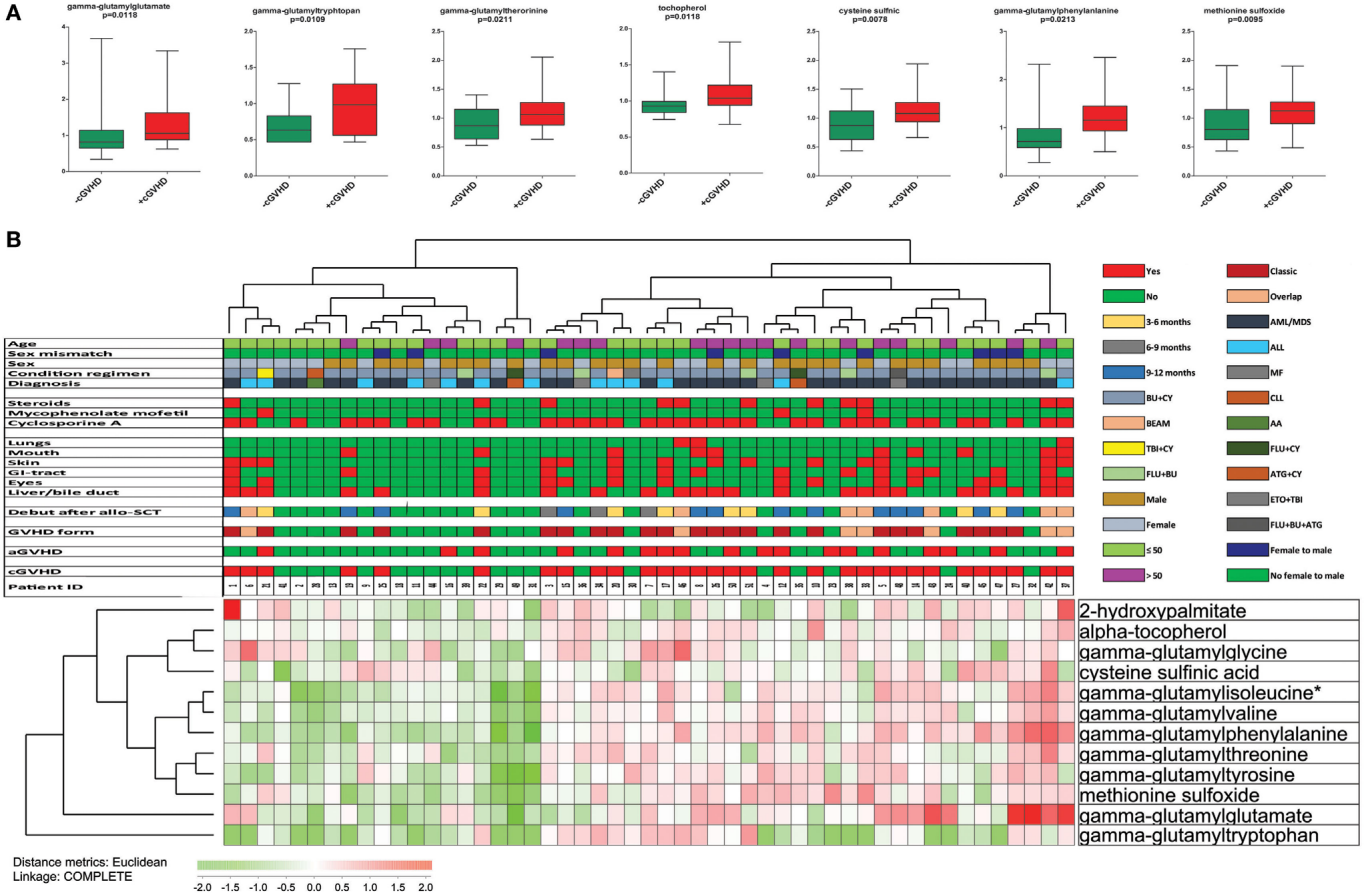
Altered serum levels of oxidative stress markers in patients with chronic graft versus host disease (cGVHD); a comparison of patients with and without cGVHD and including all 51 patients. [**(A)**, upper part] We observed increased levels of seven metabolites classified as oxidative stress markers in cGVHD patients (red boxes) compared with patients without cGVHD (green boxes). All results are presented as the median, the 25%/75% percentiles and the variation range. The metabolites and the corresponding *p*-values (Mann–Whitney *U*-test) are shown at the top of each individual figure. [**(B)**, lower part] We performed a hierarchical clustering analysis based on the 10 metabolites belonging to the group oxidative stress. The clinical characteristics of each individual patient are presented in the upper part of the figure; for the lower horizontal bars the presence of a factor is indicated by red and the absence by green, whereas the color codes for the upper horizontal bars are explained in the figure. Two main clusters could then be identified; one with generally high metabolite levels and another with generally low levels. The cluster characterized by generally high levels had a significantly higher frequency of patients with cGVHD (25 out of 32 cGVHD patients; *p* = 0.0010, Chi-square test).

Increased oxidative stress seems to be important in the pathophysiology of GVHD (33). The significantly increased levels in cGVHD patients of gamma-glutamyl amino acids (e.g., gamma-glutamylglutamate, gamma-glutamyltryptophan, gamma-glutamylphenylalanine, and gamma-glutamylthreonine) are consistent with an oxidative stress phenotype and increased activity of the gamma-glutamyl cycle that is important for recycling and regeneration of the antioxidant glutathione (34). Similarly, a significant increase in other oxidative stress markers, including alpha-tocopherol, cysteine sulfonic acid, and methionine sulfoxide (35), was also observed in cGVHD patients (Figure 5). Taken together, these observations suggest altered protein metabolism with disturbed redox homeostasis in cGVHD patients, and we therefore performed a hierarchical clustering analyses based on the 10 metabolites included in the term “oxidative stress” (Table S1 in Supplementary Material) (Figure 6). Two main clusters were then identified, and the frequency of cGVHD patients was significantly higher for the subset showing generally high levels of these metabolites (*p* = 0.0010, Chi-square test).

### Steroid Treatment of cGVHD Is Associated with Increased Levels of Phospholipids, Lysolipids, Plasmalogen, Monoacylglycerol, and Diacylglycerol Metabolites

**Figure 7:**
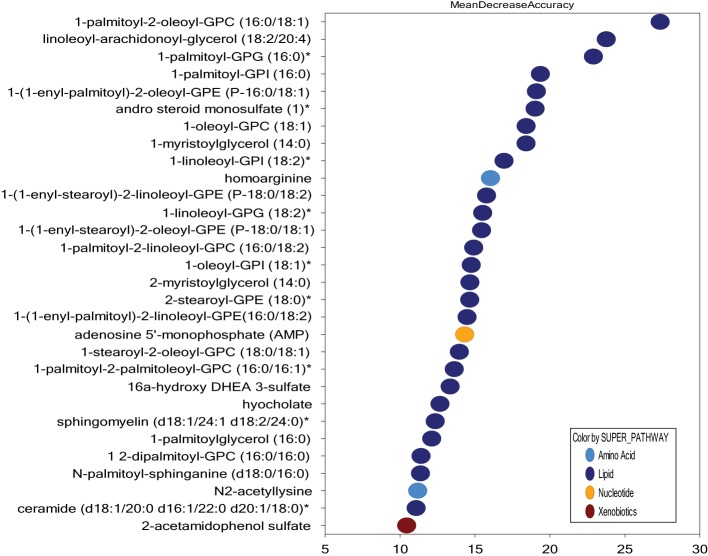
Random forest analysis based on the overall metabolomics profile for all 51 patients included in the study; a comparison of patients receiving and not receiving treatment with systemic steroids. Random forest analysis could distinguish between these two patient subsets with a predictive accuracy of 73.0%. The figure presents the 30 top-ranked metabolites and their classification (indicated in the figure, lower right) based on their importance for the identification of the two patient subsets.

We first compared the metabolic profiles for the 11 patients receiving systemic steroid therapy versus all the other 40 patients. The 30 top-ranked metabolites are presented in Figure 7. 26 of the 30 top-ranked metabolites altered by systemic steroids were classified as lipid metabolites, and 20 of these lipid metabolites belonged to the subclasses phospholipid (5 metabolites), lysolipid (7), plasmalogen (4), monoacylglycerol (3), and diacylglycerol (1). Thus, a major effect of systemic steroid treatment seems to be altered triglyceride/fatty acid metabolism.

We then did an alternative analysis and compared the metabolic profiles for the nine cGVHD patients receiving systemic steroids together with cyclosporine versus the cGVHD patients only treated with cyclosporine, i.e., comparing two patient subset with similar cGVHD/cyclosporine status but differing with regard to steroid treatment (see Figure 1 for identification of the patients in these two subsets). The top-ranked metabolites from this comparison also included a large number of lipid metabolites (20 metabolites), and most of these metabolites were classified as phospholipid (three metabolites), lysolipid (8), plasmalogen (2), monoacylglycerol (2), and diacylglycerol (1). Thus, this comparison also suggests that a major effect of systemic steroid treatment of patients with cGVHD is an altered fatty acid/triglyceride metabolism.

Finally, the steroid-associated pattern presented in Figure 7 was also reflected in our overall comparison of patients with and without cGVHD (Figure 2), although the phospholipid (2 metabolites), lysolipid (1), plasmalogen (2), monoacylglycerol (none), and diacylglycerol (none) metabolites only constituted a minor subset (5 metabolites) among the 30 top-ranked metabolites from this cGVHD comparison. These metabolites may reflect the systemic steroid treatment, but several of them are also increased during inflammation (29, 30), and alternatively reflect the more severe manifestation of cGVHD for patients requiring systemic steroids.

### Cyclosporine Has Diverse Effects on the Systemic Metabolic Profile of Allotransplant Recipients and These Effects Are Similar for Patients with and without cGVHD

**Figure 8:**
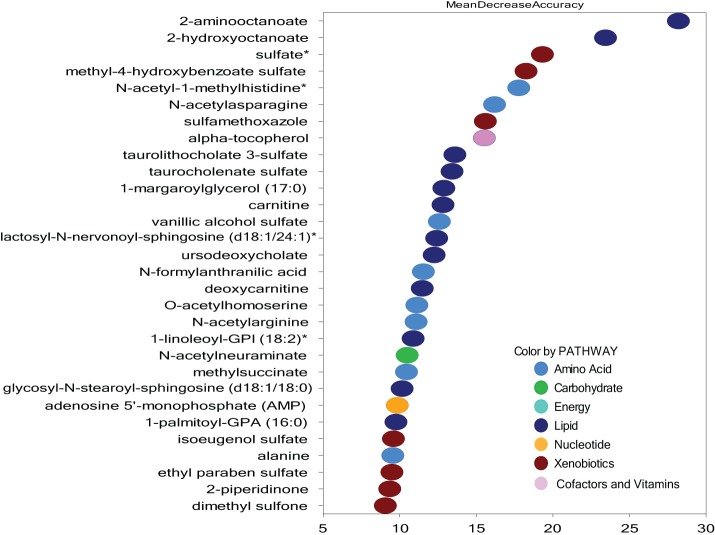
Random forest analysis based on the overall metabolomics profile for all 51 patients included in the study, a comparison of patients receiving and not receiving treatment with cyclosporine. Random forest analysis could distinguish between these two patient subsets with a predictive accuracy of 60%. The figure presents the 30 top-ranked metabolites and their classification (indicated in the figure, lower right) based on their importance for the identification of the two patient subsets.

We first compared the profile of all patients receiving cyclosporine (34 patients, including nine patients receiving combination treatment with systemic steroids) versus all the other allotransplant recipients (17 patients); the 30 top-ranked metabolites are shown in Figure 8. It can be seen that cyclosporine treatment had diverse effects and was associated with altered levels of many different metabolites/metabolite subsets. Firstly, eight of these 30 metabolites belonged to the subset amino acid metabolites, and none of them overlapped with the top-ranked metabolites for those patients receiving systemic steroids. However, 12 lipid metabolites were also included among the top-ranked metabolites; 5 of these lipid metabolites belonged to the subclasses phospholipid/lysolipid/plasmalogen and may therefore reflect alterations in the 11 cGVHD patients receiving systemic steroid, although it should be emphasized that only 3 of the 12 lipid metabolites overlapped with the 30 top-ranked metabolites identified in the comparison of patients with and without steroid treatment (Figure 7).

We then compared the metabolic profiles for a subset of patients without cGVHD and receiving no immunosuppressive treatment with the subset of seven patients characterized by without cGVHD but still receiving prophylactic cyclosporine treatment 1 year posttransplant. These subsets can be identified from Figure 1; in this subset analysis we thus could compare two groups of patients with a similar cGVHD status (i.e., no cGVHD) but differing with regard to cyclosporine treatment. Even though the ranking of individual metabolites differed, one should emphasize that 27 of the 30 top-ranked metabolites from this comparison overlapped with the 30 top-ranked metabolites identified in the previous comparison of all patients receiving cyclosporine versus all the other patients (Figure 8).

Finally we compared (1) the 30 top-ranked metabolites previously identified by the comparison of all patients with versus all without cGVHD (Figure 2) versus the (2) 30 top-ranked metabolites identified when we compared all patients with and without cyclosporine treatment (Figure 8). There was only a minor overlap including 5 heterogeneous metabolites between the 30 top-ranked metabolites identified in each of these 2 analyses, and the 5 metabolites included *N*-acetylneuraminate (amino sugar metabolism), 2-aminooctanate (fatty acid, amino), 2-hydroxyoctanoate (fatty acid, monohydroxy), lactosyl-*N*-nervonoyl-sphingosine (d18:1/24:1) (sphingolipid), and sulfamethoxazole (drug). Thus, even though cyclosporine seems to have distinct effects on the systemic metabolic profile in allotransplant recipients, the effects of cGVHD by itself seem to be stronger than the cyclosporine effects.

### Clustering Analysis Based Only on Metabolites Identified as cGVHD-Associated in Random Forest Analyses Identifies Three Patient Subsets with Different Frequencies of cGVHD

We compared the 30 top-ranked metabolites for the random forest analysis of all patients with and without cGVHD (Figure 2) and the 30 top-ranked metabolites from the analysis of all patients with and without cyclosporine treatment (Figure 8), and we then found 4 overlapping metabolites [lactosyl-*N*-nervonoyl-sphingosine (d18:1/24:1), sulfamethoxazole, 2-hydroxyoctanoate, and 2-aminooctanoate]. Similarly, when comparing the top-ranked metabolites for the cGVHD analysis (Figure 2) with the 30 top-ranked metabolites from the comparison of all patients with and without additional steroid treatment (Figure 7), we identified the five overlapping metabolites 1,2-dipalmitoyl-GPC (16:0/16:0), 1-(1-enyl-stearoyl)-2-linoleoyl-GPE (P-18:0/18:2), 1-(1-enyl-stearoyl)-2-oleoyl-GPE (P-18:0/18:1), hyocholate, and 1-palmitoyl-2-oleoyl-GPC (16:0/18:1). Thus, the 30 top-ranked metabolites from the comparison of all patients with and without cGVHD included 21 metabolites that only were associated with cGVHD but not with cyclosporine or steroid therapy.

**Figure 9:**
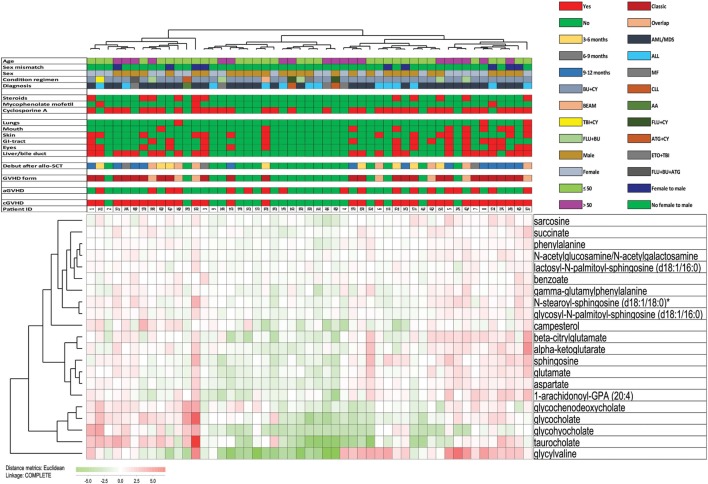
Hierarchical clustering analysis including all 51 patients and based on 21 selected metabolites. The analysis was based on the 30 top-ranked metabolites identified by the random forest analysis of the overall metabolic profile of all 51 patients and comparing patients with and without chronic graft versus host disease (cGVHD) (see Figure 2), but 9 of these 30 metabolites were excluded from this analysis because they overlapped with the 30 top-ranked metabolites identified by the comparison of patients with/without cyclosporine treatment (Figure 8) or with/without systemic steroid treatment (Figure 7). Based on the 21 remaining metabolites we performed a hierarchical clustering analysis (Euclidian Correlation, complete linkage). The heat map and corresponding dendrograms are shown in the figure together with the clinical characteristics of individual patients (see horizontal bars in the upper part). The metabolites are listed to the right in the figure; red color means high metabolite levels and green color low levels as indicated to the lower left in the figure. The clinical characteristics of each individual patient are presented in the upper part of the figure; for the lower horizontal bars the presence of a factor is indicated by red and the absence by green, whereas the color codes for the upper horizontal bars are explained in the figure.

We then did a clustering analysis of all 51 patients based on the 21 non-overlapping metabolites from the cGVHD random forest analysis (Figure 9). First, the middle cluster showed a low frequency of patients with cGVHD (3 out of 16), and this is significantly different from the other 35 patients (28/35; Chi-Square test, *p* < 0.0001). Second, patients with cGVHD were mainly included in the two other clusters (11 out of 13 in the left and 17 out of 22 in the right cluster). The frequencies of patients with cGVHD did not differ significantly between these two clusters, but the frequency of patients with extensive cGVHD affecting at least three organs was significantly higher in the right cluster (10 out of 22 patients) than in the left cluster (2 out of 13 patients; Chi-Square test, *p* = 0.027). Third, the frequencies of patients receiving cyclosporine treatment and additional steroid treatment did not differ between the right and left clusters, i.e., the two clusters including the majority of cGVHD patients. Thus, differences in immunosuppressive therapy cannot explain the localization of severely affected cGVHD patients mainly into one cluster. Finally, the two cGVHD patients without immunosuppressive therapy also clustered within the intermediate (left) cluster together with several other cGVHD patients.

Seven exceptional patients without cGVHD clustered together with the majority of cGVHD (Figure 9, left and right patient clusters). Five of these exceptional patients (patients 2, 4, 24, 32, and 41) had a minor increase in liver enzymes at the time of blood sampling without other signs of cGVHD, but additional diagnostic procedures were not indicated. Thus, they were classified as not having treatment-requiring GVHD in our study; the two last exceptional patients had no signs of cGVHD.

The three patient clusters were separated mainly due to the variation of 11 metabolites that clustered together in the lower metabolite cluster and included 5 amino acid metabolites (beta-citrylglutamate, alpha-ketoglutarate, glutamate, aspartate, and glycylvaline) and 4 bile acid metabolites (glycochenodeoxycholate, glycocholate, glycohyocholate, and taurocholate) together with the 2 additional lipid metabolites sphingosine and 1-arachidonoyl-GPA (20:4).

## Discussion

Graft versus host disease is characterized by immune dysregulation/deficiency, organ damage, and decreased survival (8, 9). Alloreactive T-cells have been implicated in its pathogenesis, but the precise role of specific T-cell subsets, autoantigens, alloantigens, and B-cells as well as the contribution from immunoregulatory soluble mediators is not known (8, 9, 20–22). Thus, GVHD reflects an exaggerated response of inflammatory mechanisms that involve donor T cells as well as multiple innate and adaptive cells and various mediators. Moreover, the involvement of inflammatory and profibrotic cytokines, such as transforming growth factor beta or platelet-derived growth factor receptors, are also important for GVHD-targeted organ injury (8, 9, 36).

We decided to investigate the patients 1 year posttransplant. This time point was selected because the impact of pretransplant and early posttransplant factors on the metabolic profile was then expected to be low, the early hematological and immunological defects in reconstitution would be less important, a substantial number of patients would have developed cGVHD but the impact on the metabolic profiles from more severe organ failures was expected to be limited.

The present metabolomic profiling study was conducted to identify serum metabolic changes associated with cGVHD. To the best of our knowledge, this is the first study to investigate the metabolic profile of patients with cGVHD. Our results have to be interpreted with care because we investigated a relatively small group of patients, but the patients are relatively homogenous because all patients received grafts from matched family donors, most of them received the same GVHD prophylaxis and a limited number of conditioning treatments were used. Our study should also be regarded as population-based because our patient cohort represents all allotransplanted patients with a family donor from a defined geographic area and during a defined time period. Additional studies are therefore needed to investigate whether our results are representative also for other subsets of allotransplant recipients (e.g., other donors).

We first compared all patients with and all patients without cGVHD, and this comparison suggests that cGVHD patients have a unique metabolic signature (Figure 2). We examined the patients at a defined time point and the metabolic profile of our patients may therefore be influenced both by differences in immunosuppressive treatment, different duration of cGVHD and thereby also differences in cumulative effects by the ongoing pathological process. Despite this heterogeneity our random forest analysis could distinguish between patients with and without cGVHD with a predictive accuracy of 75%. The identification and validation of biomarkers in cGVHD remain very challenging (10–12), but our study suggests that metabolic markers may become useful in these patients.

As stated above, the altered metabolic profile in our cGVHD patients can be caused either by the disease itself or its treatment, i.e., cyclosporine and/or systemic steroids. Several observations suggest that cyclosporine can affect systemic metabolic profiles. First, we analyzed all the 51 patients in our patient cohort and compared all patients with and all patients without cyclosporine treatment (Figure 8); the 30 top-ranked metabolites from this comparison showed a minimal overlap (only four metabolites) with the 30 top-ranked metabolites from the comparison of all patients with versus all patients without cGVHD (Figure 2). Second, to further identify metabolic effects probably caused by cyclosporine treatment we compared our patient subset without cGVHD and still receiving cyclosporine with another subset also being without cGVHD but not receiving cyclosporine, i.e., these two subsets had similar cGVHD status and differed only with regard to cyclosporine treatment. The 30 top-ranked metabolites from these two comparisons of patients with and without cyclosporine treatment showed a large degree of overlap (27 metabolites). Thus, both these comparisons suggest that cyclosporine has diverse effects on systemic metabolic profiles, and our present observations are consistent with previous observations in kidney transplant recipients (37). We conclude that cyclosporine treatment can alter systemic metabolic profiles in allotransplant recipients, but our observed differences between patients with and without cGVHD cannot be explained by cyclosporine alone because the 30 top-ranked cGVHD-associated metabolites (Figure 2) included only four of the cyclosporine-associated metabolites (Figure 8).

We used the same strategy as for cyclosporine when we investigated the contribution from steroid treatment. First, we examined the whole patient cohort and compared all patients with and all patients without steroid treatment. When comparing the results from this analysis (Figure 7) with the results from the with/without cGVHD comparison (Figure 2), we identified five overlapping metabolites, i.e., lipid metabolites associated both with cGVHD and systemic steroid therapy. Taken together these observations suggest that the increased levels of these overlapping metabolites in cGVHD patients are mainly due to the steroid treatment rather than the cGVHD. Our present results are consistent with previous studies of steroid-treated myasthenia gravis patients showing that steroids alter triglyceride/fatty acid metabolism (38). However, one cannot exclude the possibility that even these steroid-associated effects may be at least partly due to the more severe and thereby steroid-requiring cGVHD of these patients. This last possibility is actually supported by previous observations suggesting that increased levels of these lipid metabolites are also associated with inflammation (29, 30).

The altered levels of bile acid as well as tyrosine and phenylalanine metabolites in cGVHD patients may reflect at least partly an altered microbiome composition. Previous studies have demonstrated the complex and multidirectional interactions between inflammation, microbiota, and immune reconstitution in allotransplant recipients (39–43). Allo-HSCT can alter the intestinal flora and this may then be more pronounced in individuals with cGVHD (39–41). The human gut microbiome is involved in vital biological functions, such as maintenance of immune homeostasis, modulation of intestinal function and metabolic regulation; disturbances of the intestinal microbiota can thereby be associated with development and progression of inflammation, including GVHD (44). The microbial intestinal flora is responsible for the generation of several metabolites derived from amino acids, bile acids, heme, and dietary sources; several of these metabolites are absorbed and can bind specific receptors on host cells. The metabolism of aromatic amino acids, phenylalanine, and tyrosine is partly due to enzymes encoded within the microbiome (44). Thus, a change in microbiome-derived metabolites might be due to a shift in the flora with translocation of pro-inflammatory metabolites or bacterial components into the systemic circulation and thereby further acceleration of GVHD through the release of pro-inflammatory cytokines such as tumor necrosis factor-alpha and IL-1 (45, 46). Thus, our present observation supports the hypothesis that effects of altered microbiota on the systemic metabolic profile contribute to the biological and clinical impact of microbiota in allotransplant recipients (39–43).

Chronic graft versus host disease was associated with increased levels of primary bile acid metabolites. Bile acids are derived from cholesterol in the liver and released into the small intestine to facilitate dietary lipid absorption. Differences in serum bile acid levels may be caused by altered synthesis, release, or reabsorption. Bile acid malabsorption has previously been reported in GVHD (47), but an altered gut microbiome is an alternative explanation as discussed earlier. The increased levels of bile metabolites may then induce hepatic cell dysfunction and induction of pro-inflammatory mediators (48).

Serum levels of multiple markers of inflammation and oxidative stress were increased in our cGVHD patients (36), possibly reflecting an increased risk of inflammatory complications after allo-HSCT. Uremic toxicity, metabolic acidosis, and pro-inflammatory soluble mediators may activate protein degradation in cGVHD (25, 32, 49), and cGVHD may thereby be associated with altered metabolic and endocrine functions of several organs (8, 31, 36). An altered balance between protein synthesis and catabolism may then be the final result.

The altered lipid profiles in cGVHD may be due to differences in membrane lipid turnover. Immunocompetent cells switch from resting to activated state after stimulation, and this requires increased energy metabolism to fuel cell proliferation and acquire effector functions (50, 51). Disruption of lipid synthesis can reduce GVHD in murine models (52), indicating an important role of lipid metabolism in the pathogenesis of GVHD. Our findings are in concordance with these observations.

Based on our overall results we did a final clustering analysis of our entire patient cohort to distinguish between cGVHD-associated and treatment-associated metabolic effects in our patient cohort. Because we analyzed a relatively small number of patients, this analysis was based on the 30 top-ranked metabolites from the initial with/without cGVHD analysis (Figure 2). We then excluded from these 30 metabolites the 9 overlapping metabolites identified by the comparisons of patients with/without cyclosporine (Figure 8, four metabolites) and patients with/without steroid (Figure 7, five metabolites). Thus, this analysis was based on 21 cGVHD-associated metabolites and included all patients in our cohort. We did not leave out from the analysis those metabolites that may be associated with an altered gastrointestinal microbiome because we regard the microbiome as a part of the overall clinical status of allotransplant recipients. The results from this last clustering analysis (Figure 9) showed that the patients were distributed in three main clusters; one cluster included mainly patients without cGVHD, a second cluster included mainly patients with cGVHD, but the disease involved only one or two organs for the large majority of these patients; and a third cluster including a majority of patients with cGVHD and many of them having involvement of at least three organs. This identification of three patient subsets could not be explained by pharmacological differences, and it was mainly caused by differences in the systemic levels of five amino acid and six lipid metabolites (most of them being bile acids) that clustered together in Figure 9 (lower metabolite cluster). Finally, our two cGVHD patients not receiving immunosuppressive therapy clustered within the intermediate (left) cluster together with several other cGVHD patients; this observation further supports our view that this clustering analysis reflects differences in cGVHD-induced metabolic alterations.

To the best of our knowledge, this is the first study of systemic metabolic profiles in allotransplant recipients. We describe altered metabolic profiles for patients with treatment-requiring cGVHD, and the overall profile includes effect due to both cGVHD itself and the immunosuppressive treatment. However, our study identified a subset of 11 metabolites that seem to reflect both the diagnosis and the severity of cGVHD.

## Ethics Statement

This study was carried out in accordance with the recommendations of local Ethics Committee (Regional Ethics Committee III, University of Bergen, Norway; REK). All subjects gave written informed consent in accordance with the Declaration of Helsinki. The protocol was approved by the Regional Ethics Committee III, University of Bergen, Norway; REK.

## Author Contributions

HR carried out the analyses, made the data for presentation, and wrote the manuscript. I-SG, KM, and RL collected the clinical data. KH participated in the study and helped to draft the manuscript. ØB planned and organized the study, collected the data and patient informed consent, coordinated the work, and wrote the manuscript. All the authors have approved the version for publication.

## Conflict of Interest Statement

The authors declare that the research was conducted in the absence of any commercial or financial relationships that could be construed as a potential conflict of interest.

## Abbreviations

aGVHD, acute graft versus host disease; allo-HSCT, allogeneic hematopoietic stem cell transplantation; cGVHD, chronic graft versus host disease; GC, gas chromatography; G-CSF, granulocyte-colony stimulating factor; GVL, graft-versus-leukemia; HLA, human leukocyte antigen; LC, liquid chromatography; MS, mass spectrometry; PBSC, peripheral blood stem cell; PCA, principal component analysis; RIC, reduced intensity conditioning; TNF-α, tumor necrosis factor-alpha.
